# New linear solvation energy relationships for empirical solvent scales using the Kamlet–Abboud–Taft parameter sets in nematic liquid crystals

**DOI:** 10.1039/c8ra03701a

**Published:** 2018-06-21

**Authors:** Amid Ranjkesh, Meisam Hagh Parast, Olga Strzeżysz, Mohammad Sadegh Zakerhamidi, Tae-Hoon Yoon

**Affiliations:** Department of Electronics Engineering, Pusan National University Busan 46241 Korea thyoon@pusan.ac.kr; Research Institute for Applied Physics and Astronomy, University of Tabriz Tabriz Iran Zakerhamidi@tabrizu.ac.ir; Institute of Chemistry, Military University of Technology 2 Urbanowicza St. 00-908 Warsaw Poland

## Abstract

The practical application of liquid crystals (LCs) as anisotropic and ubiquitous solvents is undoubtedly lucrative. Therefore, defining solvent polarity parameters as demonstrating the effects of anisotropic LC media on the photo-physical behavior of solute molecules is increasingly sought to determine their suitability for specific areas. For this fundamental reason, a spectroscopic method was used *via* Kamlet–Abboud–Taft (KAT) polarity functions to determine the solvatochromic polarity (SP) parameters for different LCs regarding high and low dielectric anisotropy (Δ*ε*) at different temperatures and LC phases, both isotropic and anisotropic. According to empirical solvent polarity parameters, our LCs were categorized as a dipolar hydrogen bonding donor solvent. Moreover, typical and overall matrix anisotropy polarity parameters as variations of the SP parameter values between the isotropic and anisotropic phases were sorted according to Δ*ε* magnitude. Finally, we introduced the linear solvation energy relationships of empirical solvent scales with the KAT parameters sets for the first time in nematic LCs with the well-established correlations.

## Abstract 2

A new linear solvation energy relationships of empirical solvent scales with the Kamlet–Abboud–Taft parameters sets for nematic liquid crystal.

## Graphical abstract

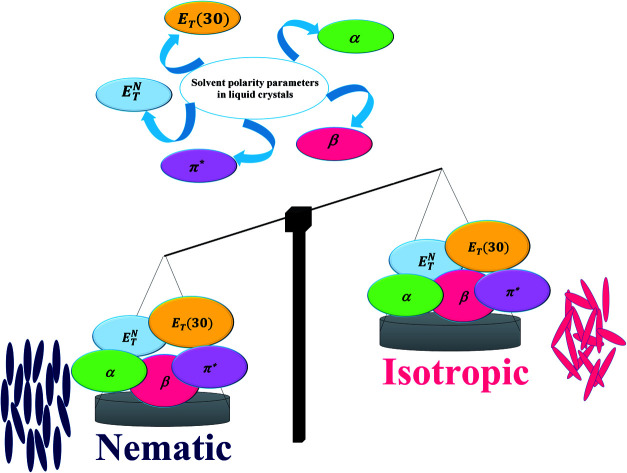

## 1 Introduction

The use of liquid crystals (LCs) as a solvent was first introduced by Svedberg^1^ in 1916. Since then, LCs have been used as compatible solvents in various applications such as optical absorption spectroscopy,^2^ gas–liquid chromatography (GLC),^3^ nuclear magnetic resonance (NMR),^4^ photoactive media,^5^ and as reaction media for thermal and photochemical reactions.^6,7^ The use of LCs as solvents has progressed in recent years because of their unique and peculiar properties.^8–11^ In LCs, the crystal lattice is relatively destroyed at certain temperatures to create a turbid liquid; then, at higher temperatures, a transparent liquid appears. This is a reversible phenomenon. Therefore, LCs represent a fascinating state of matter that lies somewhere between the solid and liquid states, whereas unlike normal isotropic liquids, which retain a wholly random molecular order, the LCs are significantly ordered.^11–13^ Furthermore, the ordering of the LC phase is somewhat limited in dissolving solute molecules where the solute molecules can be merged into the LC phase without disturbing the LC order if the structures of the solute and solvent molecules are compatible. This solute–solvent behavior is commonly connected to the term of polarity as the capability of a solvent for solvating dissolved charged, neutral, apolar, or dipolar species,^14,15^ and is associated with the function of intermolecular forces between the solvent and solute molecules. Because of insufficient simple physical solvent constants and a lack of reliable and collective theoretical expressions to determine the solvent polarity effects, the first real empirical solvent polarity parameters were introduced by Winstein *et al.*^16^ in 1948. Accordingly, the solvent polarity is appropriately defined by molecular-microscopic empirical solvent parameters, which is derived from sufficiently strong solvent-dependent model processes with individual solvent molecules.^14^ In fact, for a deep understanding of solute–solvent interactions, selection of an adequate solvent-sensitive reference process, and subsequent derivation of the empirical polarity parameters from it are crucial and significantly better descriptors compared to single physical constants of the solvent such as dielectric constant, dipole moment, and refractive index.

In past years, intensive research efforts have been devoted to determine the solvatochromic polarity (SP) parameters for commercial organic solvents, polymers, ionic liquid, and aromatic hydrocarbon.^15–20^ Moreover, to define solvent polarity scales, various measurement methods have been introduced, such as spectroscopic properties,^21,22^ equilibrium constant,^23^ kinetic rate constants of chemical reactions,^24^ and multi-parameter approaches.^25,26^ Among these measuring techniques, the spectroscopic method is based on a well-known and easily measurable experiment with solvent sensitive standard probes that dates back to 1922; Hantzsch later termed this phenomenon solvatochromism.^27^

Indeed, the solvatochromic measuring method employs the maximum absorption wavelength spectrum, which is situated within the visible region of the electromagnetic spectrum,^28,29^ by using solvatochromic dye as an indicator, which was introduced by Brooker *et al.*^30^ as the solvent-sensitive standard compound. Later, in 1958, the first systematic solvent standard was established by Kosower as a probe of solvent polarity.^31,32^ Then, several dyes in various inorganic solids, polymers, were studied by Spange *et al.*^33^ on the basis of a correlation analysis of the UV-visible spectral data. Subsequently, Spange *et al.*^34^ endorsed using the empirical polarity parameters as suitable scales in many materials.

Amongst the various scales proposed in the past,^35–37^ the Kamlet–Abboud–Taft (KAT) method^38–40^ deserves particular recognition. It is founded on the averaged spectral behavior of solutes, instead of the spectral data of any single compound. The solvatochromic comparison method was presented to explain specific and nonspecific interactions. As a result, this approach was developed to comprise a solubility parameter for microscopic and macroscopic quantities. Four parameters of the KAT solvent scales were introduced and defined: *E*^N^_T_, the normalized solvent polarity parameter; *α* and *β*, which give a quantitative measure of the hydrogen-bonding capabilities of a solvent's hydrogen bond donor (HBD) acidity and hydrogen-bond acceptor (HBA) basicity, respectively; and finally, π* as a measure of the solvent's dipolarity/polarizability.^38–43^ There are very few reports on determining the KAT parameters at the different LC phases. For example, Sıdır *et al.*^41^ measured these parameters for the absorption and fluorescence spectra of some LC derivatives in organic solvents. Ichikawa *et al.*^42^ studied the linking between the polarity of amino acid ionic liquids with the lyotropic LC system. Undeniably, the investigations of the SP parameters for the new nematic LCs or mesogenic compounds in negative or positive dielectric anisotropy (Δ*ε*) with their magnitudes for defining quantitative SP parameters are essential and notable achievements because of their practical uses in different technological device applications and chemical and biological systems.^11,13^ Consequently, determining the SP parameter values for various LCs regarding various phases, temperatures, and variations of nematic-isotropic states open a new window for introducing the new parameters for LCs, as well as at different phases. In particular, in the isotropic phase and pre-transitional temperature region, most physical parameters completely disappear; therefore, the SP parameters can introduce and help us in this region by quantitative values. Beyond these achievements, a profound investigation of the LC properties comprehensively expands our knowledge regarding the LC features to improve the synthesize of new LCs for practical applications and related industry.

In this work, we quantitatively determined and characterized the solvent polarity parameters for three unknown molecular mixture LCs at different temperatures and phases, isotropic and anisotropic, using the solvatochromic method. For our investigation, three LCs were selected: one with a negative Δ*ε* value, one with an intermediate positive Δ*ε* value, and one with a quite high Δ*ε* value. The SP parameters were determined in the nematic phase, phase transition area, and isotropic phase. In the isotropic phase, where the most macroscopic physical parameters vanish completely, the SP parameters can provide a physical evidence with the quantitative values. Finally, in the first time, the linear solvation energy relationships of the empirical solvent scales for the nematic LCs are introduced by using the calculated SP parameters. The solvent polarity scales facilitate the systematic correlation and analysis of chemical and physicochemical properties in the LC solution media. These considerations lead one to think positively about using LC media as suitable solvents in selective areas.

## 2 Experimental

### 2.1 Materials

We procured and used spectroscopy-grade solvent-sensitive standard dyes and coumarin 504 from Sigma-Aldrich (Taufkirchen, Germany) without further purification. In addition, an azo dye (DR_2_), used as a probe, was synthesized and purified in our laboratory.^21,22^ All solvatochromic indicators are listed in Table 1. Three different LCs with different positive and negative Δ*ε* were used to compare their SP parameters. We employed LC-I (ML-1407, Δ*ε* = −4.1, Merck Ltd) as the low and negative Δ*ε*, LC-II (MAT-16-968, Δ*ε* = 15.8, Merck Ltd.) as positive and intermediate Δ*ε*, and finally LC-III (LC-2082, Δ*ε* = 41.7) as the high and positive Δ*ε*, which was synthesized at the Institute of Chemistry of the Military Technical Academy in Warsaw, Poland for our studied LCs. Table 2 shows detailed information of the used LCs.

**Table 1:** Solvatochromic indicators and dyes used in the experiments

Dye structural name	Molecular structure and structural name
*N*,*N*-Dimethyl-*p*-nitroaniline	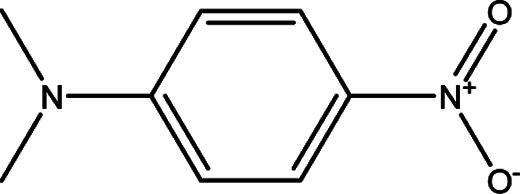
*p*-Nitroaniline	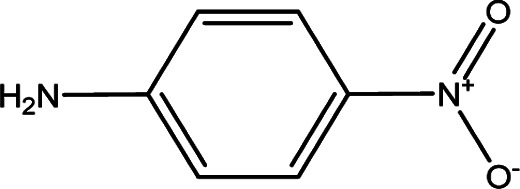
Reichardt's betaine dye	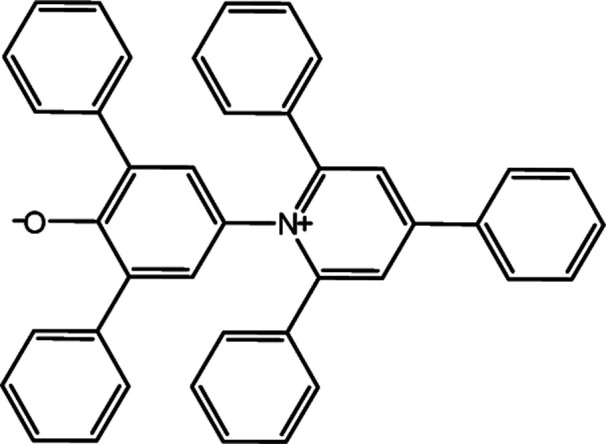
2,6-Diphenyl-4-(2,4,6-triphenyl-1-pyridinio)phenolate	
Coumarin 504	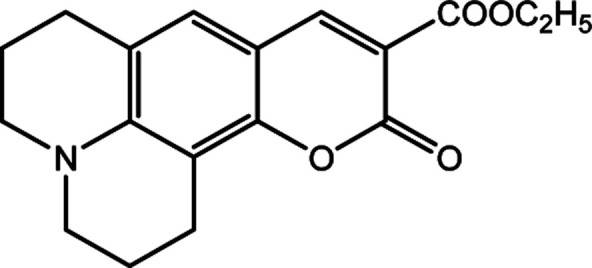
2,3,5,6-1*H*,4*H*-Tetrahydro-9-carbethoxyquinolizino-[9,9*a*,1-*gh*]coumarin	
DR_2_	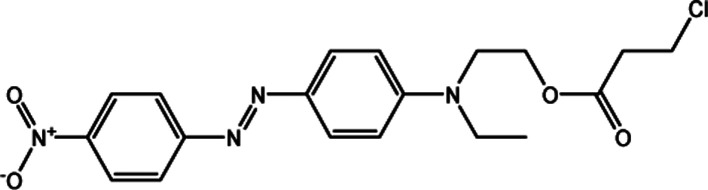
4′-Nitro-4-(*N*-ethyl,*N*-2-(3-chloropropanoyloxy)ethyl azobenzene	

**Table 2:** Material parameters of four nematic liquid crystals: phase transition temperature (*T*_c_), mean dielectric constant (*ε̄*), dielectric anisotropy (Δ*ε*), and birefringence (Δ*n*)

Liquid crystal	*T*_c_/°C (±0.1)	*ε̄* (1.0 kHz, ±0.01)	Δ*ε* (±0.01)	Δ*n* (589 nm, ±0.001)
LC-I (ML-1407)	75.5	6.46	−4.10	0.101
LC-II (MAT-16-968)	72.7	12.00	15.80	0.187
LC-III (MLC-2082)	89.9	20.60	41.70	0.180

### 2.2 Absorption spectroscopy

After making an extremely dilute solution of polarity indicator dye, (10^−5^ M), the absorption spectra were measured over a wavelength range between 300 and 800 nm using a double-beam UV-2450 scan spectrophotometer (Shimadzu Corp., Tokyo, Japan). The cell temperature was controlled with a circulating water bath while checking the precise temperature (±0.1 °C), particularly near the phase transition region.

### 2.3 Liquid crystal cell preparation

We fabricated an LC cell using two sandwiched optical glass plates (2 × 1.2 cm^2^) with Mylar film (12.5 μm) as a cell spacer, then sealing the glass plates together with epoxy resin glue. Finally, the solution of the selected nematic LC with standard indicator dye was filled into the cell *via* capillary action.

### 2.4 Determination of the solvent polarity parameters by the solvatochromic method

The KAT parameters were determined by the solvatochromic method using the wavenumber of the maximum absorption of each indicator, expressed in kK (kilokeyser, 1000 cm^−1^). Parameter π* delivers a measure of a solvent's dipolarity/polarizability ratio. We employed cyclohexane and dimethyl sulfoxide to calculate the π* value corresponding to the maximum transition frequency of a non-hydrogen-bonding solvatochromic probe in the solvent in terms of the frequency of the probe.^38,43^ The π* values were calculated using *N*,*N*-dimethyl-*p*-nitroaniline as a probe by^38,43,44^1
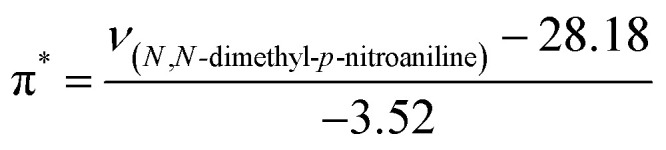


We employed an additional solvent-sensitive dye, namely the disperse azo dye (DR_2_), with absorption bands in the range from 440–510 nm, because of the overlap between the maximum wavelength of the absorption band of the LCs and *N*,*N*-dimethyl-*p*-nitroaniline, as well as with other π* indicators in the UV region, to situate the absorption band located in the visible region.^21,22,43^ Therefore, the linear correlation between the maximum wavelength of DR_2_ and *N*,*N*-dimethyl-*p*-nitroaniline in organic solvents was obtained to determine the maximum wavelength of π* in anisotropic media by^21,22^

2 *ν*_(*N*,*N*-dimethyl-*p*-nitroaniline)_ = 1.19*ν*_(DR_2_)_ + 594.39, *R*^2^ = 0.98

Parameter *β* provides a measure of a solvent's HBA basicity. The *β* parameter is obtained by measuring the relative difference of solvatochromism between the wavenumber of the longest wavelength of the absorption band of the *p*-nitroaniline and *N*,*N*-dimethyl-*p*-nitroaniline dyes by^40,45,46^3
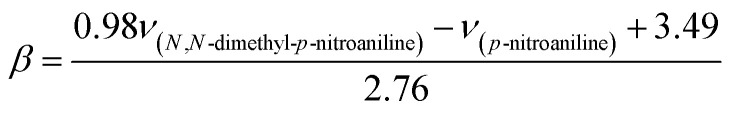


For the maximum wavelength of *p*-nitroaniline in eqn (3), a similar method in the *N*,*N*-dimethyl-*p*-nitroaniline case was used because of the overlap in the maximum wavelength of the absorption band of the LCs and *p*-nitroaniline (and other *β* indicators) in the UV region, where an excellent linear correlation between the maximum wavelength of *p*-nitroaniline and DR_2_ was obtained by^21,22,43^

4 *ν*_(*p*-nitroaniline)_ = 1.69*ν*_(DR_2_)_ − 8404.40, *R*^2^ = 0.96

The *α* parameter provides a measure of a solvent's HBD acidity, and with the longest wavelength of the absorption band of Reichardt's betaine dye, its value was determined by^46^5
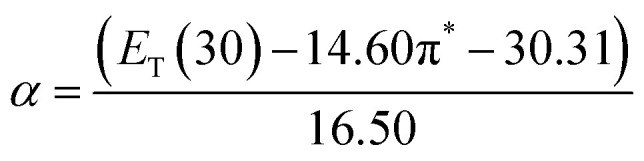


Herein, *E*_T_(30) is an empirical solvent polarity scale, where commonly a zwitterion compound, known as Reichardt betaine dye, was employed to define the polarity of solvents; 30 specifies the number allocated to this dye, and signifies the energy required to move to the excited state from the ground state. The *E*_T_(30) and scales state the solvent polarity rising from overall interactions between a solvent and the dye and is simply determined as the molar transition energy of Reichardt's betaine dye expressed in kcal mol^−1^:^1,47^6
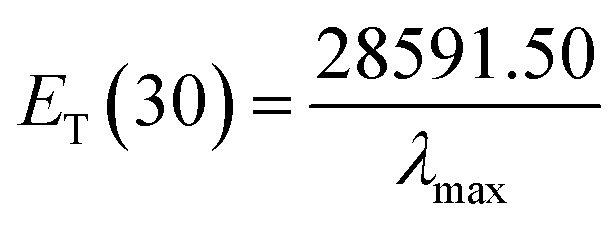
where *λ*_max_ is the maximum-absorption wavelength of the standard Reichardt's betaine dye. It is often recommended to use the normalized *E*^N^_T_ for which tetramethylsilane (*E*^N^_T_ = 0) and water (*E*^N^_T_ = 1) are introduced as extreme nonpolar and polar reference solvents, respectively. Thus, *E*_T_(30) scaled in the SI units framework as a dimensionless normalized *E*^N^_T_ scale was used:^47,48^7
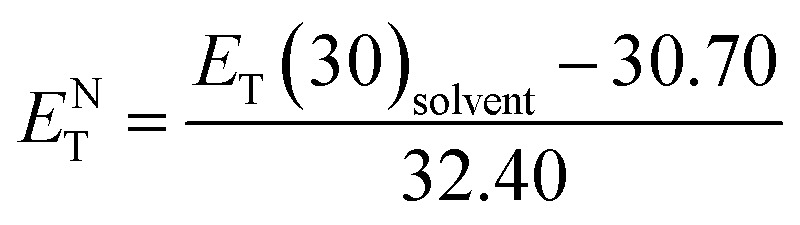
*E*^N^_T_ is a highly sensitive parameter for characterizing the polarity of solvents because of the extremely large shift of the charge transfer absorption band of the dye molecule. The *E*_T_(30) and *E*^N^_T_ scales express the solvent polarity arising from overall interactions between a solvent and the dye.

The 2,6-diphenyl-4-(2,4,6-triphenyl-*N*-pyridino) phenolate is insoluble in the investigated LCs; therefore, to determine *E*_T_(30) and *α* for these LCs, the high-solubility coumarin 504 dye was employed.^43^ The correlation between the maximum wavelength of Reichardt's betaine dye and coumarin 504 in different solvents was achieved by the following relationship:^21,22^

8 *ν*_(Reichardt's betaine dye)_ = −7.48*ν*_(504)_ + 188778.00, *R*^2^ = 0.91

## 3 Results and discussion

### 3.1 Solvatochromic polarity parameters in the nematic phase of the liquid crystal

The results of solvatochromic polarity parameters for our investigated LC media at room temperature (25 °C) are reported in Table 3. The values of π*, *α*, and *β* for LC-III show the highest magnitudes in comparison with other studied LCs because of its high Δ*ε* value as a high polar LC. The ranges of the *β* values from 0.52 to 0.58 for our investigated LCs indicate a higher HBA capacity, whereas their values are larger than a few ionic liquids containing a pyridinium cation in *β* ranges of 0.29–0.40.^48^ It is well-known after the phase transition temperature (*T*_c_), the long rang order (LRO) in the nematic phase convert to short-range order (SRO) in the isotropic phase.^11^ Therefore, the minimum *β* value in the LC-II can be explained due to the lowest LRO in the nematic phase of LC-II in comparison with other investigated nematic LCs. This behavior of LCs is totally different from the isotropic liquids which they have no ordering behaviors. For example, the *β* value for ethyl acetate, ethyltrichloroacetate, and ethyl trifluoroacetate are 0.45, 0.25, and 0.19, respectively, because exposing the high electronegative fluorine substituent decreases the *β* value.^46^ Large π* values showing the dipolarity/polarizability characteristics were observed in our LCs in comparison with the ordinary solvents, because of the inherent highly polar nature of LC media and strong interaction with dipole solutes. For a better understanding of the LCs' polarity properties, comparison of the reported SP parameters of our studied LCs with the ordinary organic solvents can provide interesting information about the obvious differences between them.

**Table 3:** Solvatochromic parameters determined at room temperature (25 °C) for studied nematic LCs

Liquid crystal	π*	*α*	*β*	*E*_T_(30)	*E* ^N^_T_
LC-I	0.50 ± 0.02	0.28 ± 0.03	0.52 ± 0.01	42.22 ± 0.01	0.36 ± 0.05
LC-II	0.46 ± 0.03	0.49 ± 0.05	0.49 ± 0.04	45.01 ± 0.05	0.45 ± 0.03
LC-III	0.62 ± 0.04	0.36 ± 0.01	0.58 ± 0.02	45.40 ± 0.02	0.45 ± 0.01

For this reason, we selected acetone as a well-known and practical organic solvent with the mean dielectric (*ε̄*) value (*i.e.*, *ε* = 20.59 at 25 °C)^49^ approximately similar to that of LC-III. As a predictable result, LC-III exhibits a low HBA. On the other hand, it has large dipolarity/polarizability groups in its structure owing to the high inherent polarity nature of LCs, particularly at its high Δ*ε* value; for LC-III, *β* = 0.58 and π* = 0.618, compared with *β* = 0.48 and π* = 0.71 for acetone reported by Kamlet *et al.*^46^ However, a comparable π* value for LC-III is that observed for fluorobenzene (π* = 0.62)^46^ as a high polar aromatic solvent. In further comparisons, the π* value of ethyl propionate (*ε* = 5.65 at 25 °C)^50^ as another practical solvent compares with our investigated LC-I, having relative dielectric constant values. The π* values for LC-I is 0.501, slightly higher than that reported for ethyl propionate (π* = 0.47) as reported by Kamlet *et al.*^46^ This result could be verified as a general declaration regarding the intrinsically polar characters of the LCs, even for those with low Δ*ε* values. The approximately similar π* values for LC-I are obtained with some polar organic solvents, such as trifluoroacetic acid (π* = 0.5), *N*,*N*-dimethylbenzylamine (π* = 0.49), and 1,1,1-trichloroethane (π* = 0.49).^46^ However, because of the unknown molecular structures of our investigated LCs, we could not state the reason for differences in the SP parameters specifically. For this reason, for better comparison of their SP parameters values, we selected the values of the SP parameters with previous reports for obvious and unknown molecular LCs structures^21,22,43^ in comparison with our investigated LCs.

In the first step, by comparison with unknown molecular structures, the similar *β* and π* values for our LC-III can be observed with MLC-2053 (Δ*ε* = 42.6) as a mixture LC reported earlier,^43^ considering the closeness of their dielectric anisotropy. In the same way, approximately similar dielectric constant values for our LC-I were observed with MLC-6292 (Δ*ε* = 7.4, *ε̄* = 6.2) with unknown molecular structures.^43^ Therefore, it could be anticipated that MLC-6292, because of the high magnitude of its *β* and π* values (*β* = 0.55, π* = 0.56), would have higher dipolarity/polarizability than our LC-I; thus, it probably has HBA groups such as –CN, –NCS, –COO, or possibly –F, in its structure. Finally, in a comparison between all our investigated LCs with 1294-1b, (a mixture LC with anonymous structure^22^) shows low *β* and π* magnitudes; subsequently, it can be concluded that our investigated LCs have low HBA and dipolarity/polarizability abilities.

In the second step, we compared the SP parameters of our investigated LCs with a few obvious LC molecular structures to determine in depth the solvent polarity behaviors in the LC media. However, it should be noted comparison of *α*, *β*, and π* values with other LCs provide the estimating power of HBA and HBD capabilities and dipolarity/polarizability characteristics in our investigated LCs. By comparison of slightly analogous values of the mean dielectric constant of LC-I (*ε̄* = 6.44) with that of 6CHBT having a –NCS functional group in its structure (*ε̄* = 6.9),^22^ it might be anticipated the low dipolarity/polarizability and HBA groups with the large HBD substituents can be found in our LC-I structure. Additionally, more comparison can be made for similar values of the mean dielectric constant of our LC-II (*ε̄* = 5.33) with MBBA (*ε̄* = 5.2),^22^ a commercially available and technically usable LC with a biphenyl structure. The results show that our LC-II may possess higher HBD capability and lower dipolarity/polarizability characteristics and HBA ability in comparison with MBBA (*α* = 0.08, *β* = 0.68 and π* = 0.8).^22^ Furthermore, it is notable that all our investigated LCs showed lower *β* and π* values in comparison with 5CB, 6CB, and E_7_,^21^ conventional LCs having a –CN functional group in their structures. Therefore, it can be concluded that having a polar functional group in the structures of all 5CB, 6CB, and E_7_ LCs caused increases in their Δ*ε* values^21^ as compared with our LC-I and -II. However, LC-III, because of its lower π* and *β* values than all 5CB, 6CB, and E_7_ LCs, may exhibit weakened dipolarity/polarizability ability even with high its Δ*ε* value.

Next, in a comparison of *α* values as an indication of HBD ability, low values for all our LCs are shown in comparison with π* and *β* values. In comparison with a few organic solvents, the *α* values for our investigated LCs were larger than that of acetone (*α* = 0.08), 2-butanone (*α* = 0.06), and acetonitrile (*α* = 0.22),^46^ but lower than that of methanol (*α* = 0.93), ethanol (*α* = 0.83), acetic acid (*α* = 1.12), and *tert*-butanol (*α* = 0.68).^46^ However, relatively comparable magnitudes can be realized with polyethylene glycol (PEG-400, *α* = 0.31),^51^ PEG-600 (*α* = 0.32),^51^ and methylene chloride (*α* = 0.30).^46^ Additionally, the values of *α* for all the studied LCs are considerably higher than those studied LCs reported earlier, including E_7_, 5CB, 6CB, MBBA, mixture 1294-1b, 7CP5BOC, and 7CP7BOC.^21,22^ Therefore, the higher *α* magnitudes in our studied LCs reveal more HBD groups in their structures in comparison with other LCs reported up to now.

Finally, the *E*^N^_T_ parameter represents a universal overall polarity scale ranging from 0.000 for the least polar solvent, tetramethylsilane (TMS), to 1.000 for the most polar solvent, water. To avoid the non-SI unit kilocalories per mole (kcal mol^−1^) and the conversion of the *E*_T_(30) values into kilojoules per mole (kJ mol^−1^), the normalized *E*^N^_T_ or *E*_T_(30) scale can be used equally. *E*_T_(30) is a descriptor of both hydrogen bond and electrostatic interactions of solvents, and large *E*_T_(30) or *E*^N^_T_ values correspond to high solvent polarity.^52^ The *E*_T_(30) scale is greatly influenced by dipole moment, polarizability, and hydrogen bonding interaction. According to the magnitudes of the *E*^N^_T_ parameter in our studied LCs, they can be classified as dipolar non-HBD solvents, where this classification corresponds to values of well-known organic solvents.^28^ Moreover, the minimum and maximum ranges of the *E*^N^_T_ parameter for our LCs are 35.4% and 45.6%, respectively, of the solvent polarity of water, the most polar solvent. The *E*^N^_T_ results for our investigated LCs show relatively comparable values with acetone (*E*^N^_T_ = 0.35), acetonitrile (*E*^N^_T_ = 0.46), and dimethyl sulfoxide (DMSO, *E*^N^_T_ = 0.444) as the most practical organic solvents.^28^

### 3.2 Temperature dependencies of the solvatochromic polarity parameters in the liquid crystal

**Fig. 1:**
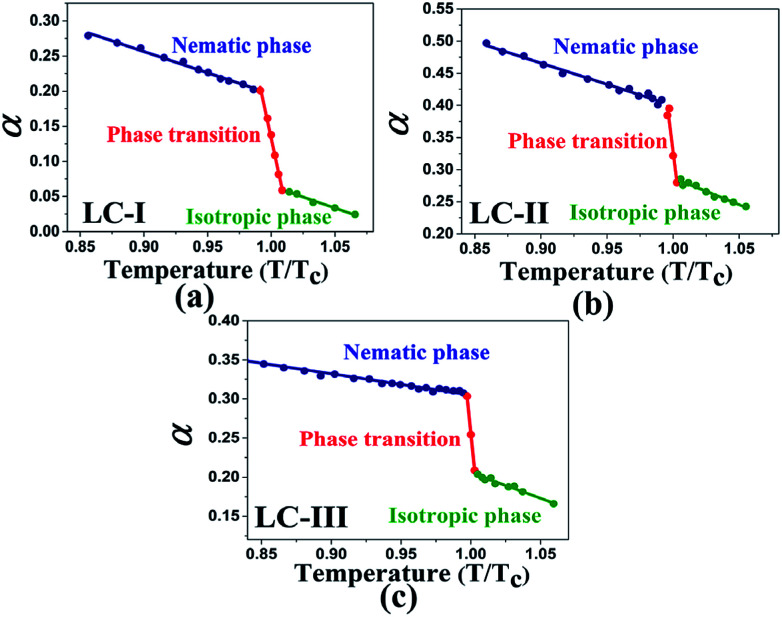
The temperature-dependent *α* parameter in the studied nematic LCs. (a) LC-I; (b) LC-II; and (c) LC-III.

**Fig. 2:**
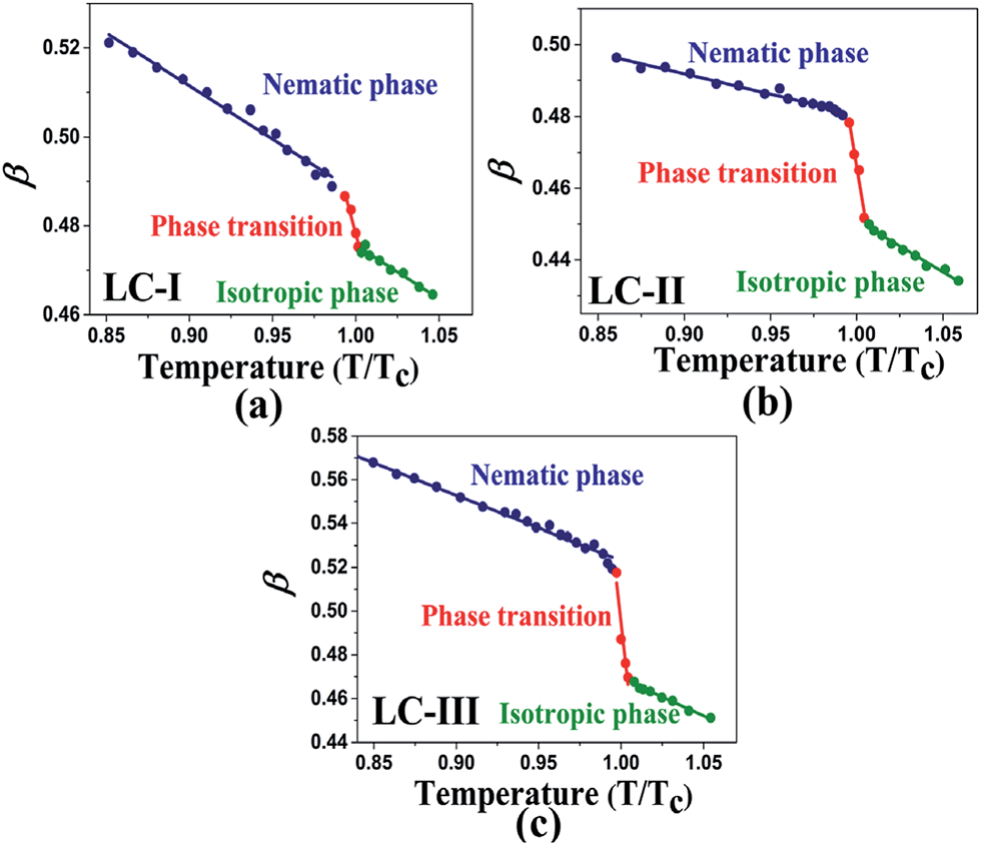
The temperature-dependent *β* parameter in the studied nematic LCs. (a) LC-I; (b) LC-II; and (c) LC-III.

**Fig. 3:**
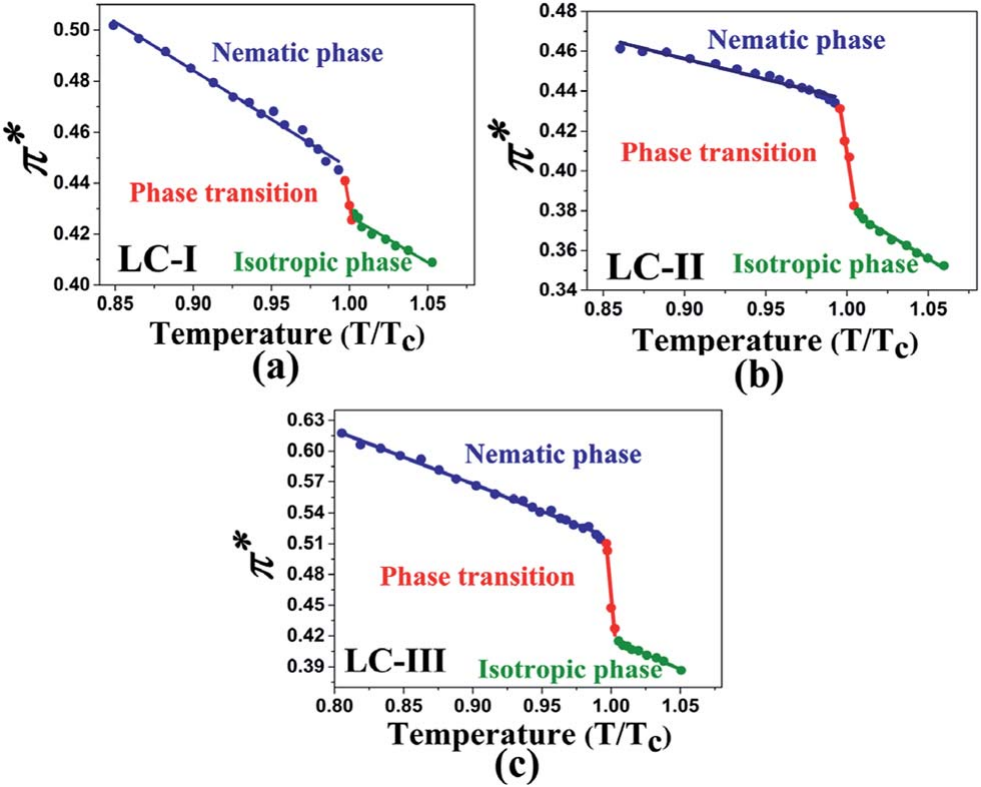
The temperature-dependent π* parameter in the studied nematic LCs. (a) LC-I; (b) LC-II; and (c) LC-III.

**Fig. 4:**
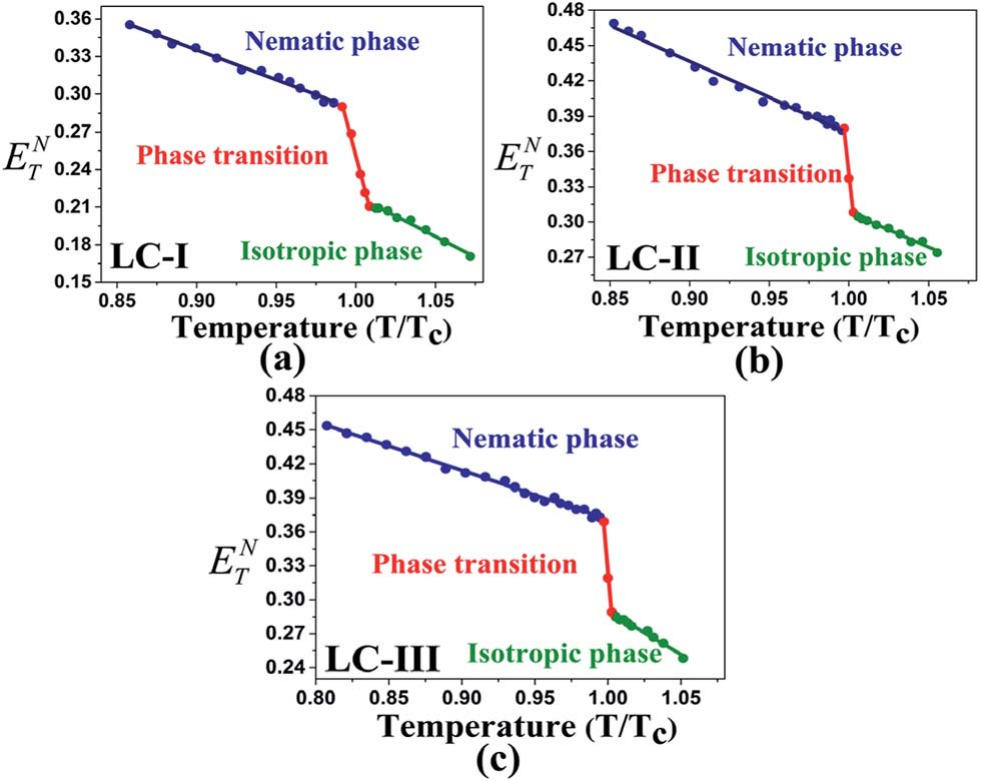
The temperature-dependent *E*^N^_T_ parameter in the studied nematic LCs. (a) LC-I; (b) LC-II; and (c) LC-III.

**Fig. 5:**
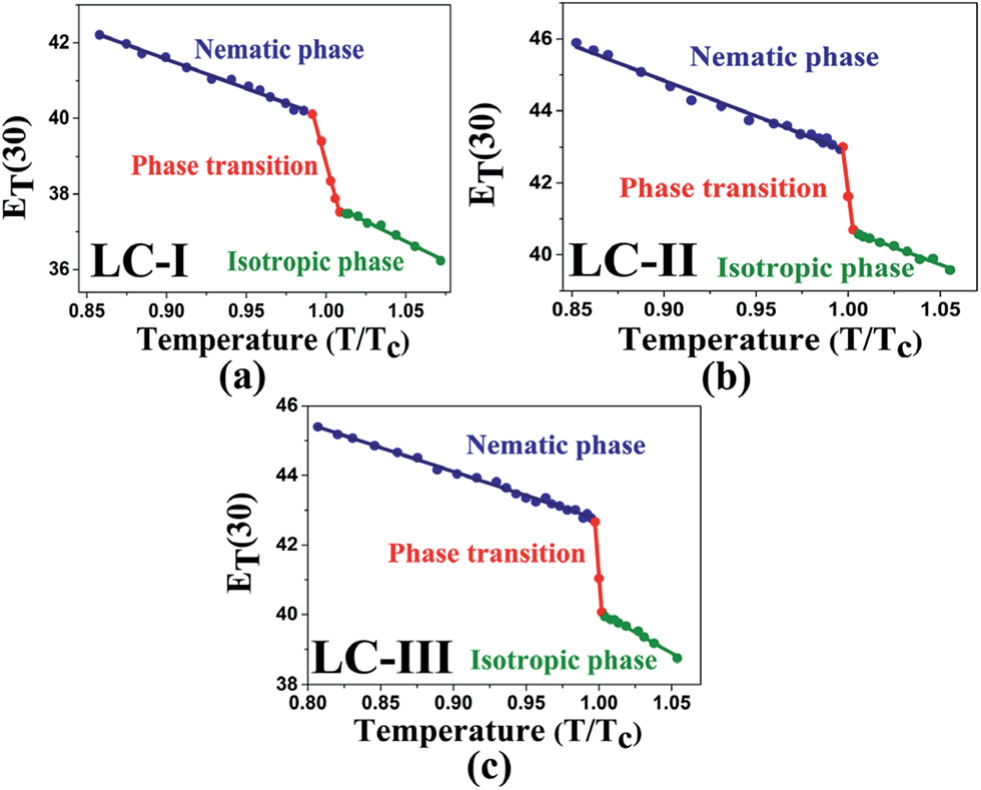
The temperature-dependent *E*_T_(30) parameter in the studied nematic LCs. (a) LC-I; (b) LC-II; and (c) LC-III.

Temperature is one of the most important factors in the success of the planned reaction to select a suitable solvent. Temperature affects the different phases of LCs; the behavior of nematic, nematic-isotropic, and isotropic states are completely different from those of ordinary solvents because of the LCs' altered interactional behavior, orientational order, rigidity, and movement of the LC media as solvents for use in various areas. Consequently, studies of the temperature dependence of solvatochromic polarity parameters at different phases are critical even in the isotropic phase, because as temperature increases, molecular thermal fluctuation and movement of LC molecules in the isotropic phase increases as compared with the anisotropic phase, and LC molecules exhibit behavior similar to that of ordinary solvent. The solvatochromic parameters were calculated by measuring the maximum of the absorption bands of the polarity indicator in three stages, considering a solvatochromic method for each temperature. The temperature variations of the SP functions in the investigated LCs are shown in Fig. 1–5, which show three distinct trends in the LCs' interaction behaviors. It was predictable that the KAT polarity functions are temperature-dependent; sharp reductions in their values can be observed with increasing temperature for all SP parameters and phases. It is notable that in the nematic phase, because of the decreasing rigidity and directional order, decreasing magnitudes of all π*, *β*, and *α*, and *E*_T_(30) values can be observed with increasing temperature. On the other hand, near the nematic-isotropic (T_N-I_) phase region, large variations are observable in all KAT parameters as shown in Fig. 1–5. The reason for this phenomenon might be associated with the large variation in directional ordering, movement of the LC molecules, and consequently changing intermolecular interactions of the LCs due to slight temperature increases.

### 3.3 Solvatochromic polarity parameters in the isotropic phase of the liquid crystal

The isotropic phase accompanies a zero-order parameter that exhibits behavior similar to that of ordinary solvent. With increasing temperature, higher thermal fluctuations and increased movement of the LC molecules occur in the isotropic phase in comparison with the nematic phase. Therefore, under an increase in temperature, sharper reductions along with larger slopes for temperature-dependent π*, *β*, *α*, *E*^N^_T_, and *E*_T_(30) values in the isotropic phase are observed in comparison to the nematic LC phase. To confirm our statement regarding the strong similarities between the isotropic phase in LCs and ordinary solvents, we compared our solvents' polarity function values (π*, *β*, and *α*) and *E*^N^_T_ in the isotropic phase with those conventional solvents, as taken from the literature. In the isotropic state, *α* value for LC-I (*i.e.*, Fig. 1(a)) shows magnitudes comparable to those of 2-butanone (*α* = 0.06).^46^ For the LC-II and -III, the *α* values show similar to acetone (5.0 MPa, 40 °C, *α* = 0.28).^51^ By comparing of *α* parameter in nematic and isotropic phases, the unchangeable position can be observed for sorting of *α* values as LC-II > LC-III > LC-I. This behavior reveals that *α* parameter is no affected by the order of nematic LCs, on the other hand the *β* parameter behave totally different in which it strongly depends on the ordering of LCs. Therefore, it can be concluded that *α* parameter behaves independently from *β* parameter.

The values of *β* in our investigated LCs in the isotropic state start from 0.44 or higher magnitudes (*i.e.*, Fig. 2(a)–(c)), which represents a higher HBA capacity in comparison with conventional and practical solvents such as dioxane (*β* = 0.37), toluene (*β* = 0.11), benzene (*β* = 0.10), and acetonitrile (*β* = 0.31), and even more than some ionic pyridinium liquids as reported by Lee *et al.*^48^ The magnitudes of *β* reported by Kamlet^46^ for diethyl ether, ethyl acetate, and di-*n*-butyl ether are *β* = 0.47, 0.45, and 0.46, respectively, and are similar to those of the isotropic phases of LCs-I, -II, and -III. On the other hand, in the same report by Kamlet,^46^ the values of *β* extracted for organic solvents such as methyl acetate (*β* = 0.42), benzonitrile (*β* = 0.4), nitrobenzene (*β* = 0.39), ethyl benzoate (*β* = 0.41), and benzophenone (*β* = 0.42) show lower values than our investigated LCs as shown in Fig. 2(b and c). However, due to highly order nature of LC at vicinity of *T*_c_, SRO in the isotropic phase of nematic LCs still remains. Because of changing these orders, the SP parameters in the LCs are shown different values and behaviors from nematic to isotropic phases. Sequences of *β* values in the nematic phase are sorted as LC-III > LC-I > LC-II, on the other hand in the isotropic phase they arranged as LC-I > LC-III > LC-II. In the same comparison for π* in the nematic phase of investigated nematic LCs are organised as LC-III > LC-I > LC-II, on the other hand in the isotropic phase is arranged as LC-I > LC-III > LC-II. From *β* and π* results, the positions of LC-I and -III are exchanged to each other from nematic to isotropic phases, but for LC-II the lowest magnitudes are remained in the both conditions. It can be concluded in the LC-II, SRO is really weak in comparison with other nematic LCs. This result confirms that changing of ordering LCs in two distinct phases can subsequently alter the behavior of SP parameters.

The comparison of π* values in the isotropic state of our studied LCs (*i.e.*, Fig. 3(a)–c)) with those of other isotropic solvents indicates lower magnitudes than those of some organic solvents, including acetic acid (π* = 0.50), chloroform (π* = 0.58), ethanol (π* = 0.54), and methanol (π* = 0.60);^46^ on the other hand, similar values with two alcohol solvents, *t*-butanol (π* = 0.41) and 1-pentanol (π* = 0.42)^46^, can be observed with LC-I and -III as shown in Fig. 3(a) and (c). For the isotropic phases of LC-II, the π* values are determined similar to eucalyptol (π* = 0.36) as reported by Laurence *et al.*^53^

The low *E*_T_(30) or *E*^N^_T_ values in the isotropic phases of LC-I, -II, and -III correspond to the low solvent polarity and the electrostatic interactions in comparison with some alcohol solvents with hydrogen bonding interaction, such as methanol (*E*^N^_T_ = 0.762 and *E*_T_(30) = 55.4) and ethanol (*E*^N^_T_ = 0.654 and *E*_T_(30) = 51.9).^14^ However, the *E*^N^_T_ value in the initial point of isotropic phase in the LC-I; the lowest values among of our LCs, exhibits a higher value than that of fluorobenzene (*E*^N^_T_ = 0.194), an important polar solvent.^14^ Thus, this result confirms our statement regarding the inherent highly polar nature of the LC media even in the isotropic state to interact strongly with dipole solutes. One more comparison can be made for approximately similar *E*_T_(30) and *E*^N^_T_ values for LC-III in an isotropic state as depicted in Fig. 4(c) and 5(c) with chloroform (*E*_T_(30) = 39.1 and *E*^N^_T_ = 0.259) as reported by Reichardt.^14^

One practical use of *E*_T_(30) or *E*^N^_T_ parameters can be stated in the pre-transitional and isotropic regions. Above T_N-I_ of nematic LCs, most electrical and optical anisotropic quantities of the macroscopic physical parameters (*i.e.*, Δ*n* and Δ*ε*) originating from the long-range ordering behaviors completely disappear; on the other hand, the short-range ordering between the nematic LC molecules still exists.^13,14^ Two parameters describing the electrostatic intermolecular interactions, so-called *E*_T_(30) or *E*^N^_T_ with quantitative values, endorse the presence of the short-range interaction in those regions appropriately.

### 3.4 Solvatochromic polarity parameters in the phase transition from nematic to isotropic phases of the liquid crystal

As shown in Fig. 1–5, highly sensitive variations between the temperature dependence of the SP functions occurred in the isotropic and anisotropic phases of the LCs, whereas for ordinary solvents, linear trends can be observed with little variation.^14^ Consequently, the variations between two phases can be prepared for crucial and valuable information for the LCs' behaviors for practical application of the LCs as solvents. For this reason, introducing an overall characterization of typical matrix anisotropy, signifying the variation between polarity functions in two isotropic and anisotropic phases of LCs, helps to determine the details of the LCs' SP features.^21,22,43^ The typical *Z* parameters values were determined for investigated LCs from the variation of SP parameters values between nematic and isotopic phases. In other words, *Z* values can be determined by subtracting values in the last point before *T*_c_ and the first point after *T*_c_ of each SP values by assisting of Fig. 1–5. Accordingly, the typical interactions of dipolarity/polarizability (*Z*_π*_), HBA (*Z*_*α*_), and HBD (*Z*_*β*_) ability are characterized for the solvent polarity functions including π*, *β*, and *α* matrix anisotropy effects, respectively. Meanwhile, the temperature dependence variations of *E*_T_(30) and *E*^N^_T_ are linked to the value of overall matrix anisotropy as *Z*_o_, and *Z*^N^_o_, respectively, considering the overall solvent polarity parameter functions. Results of *Z*_o_, *Z*^N^_o_, *Z*_π*_, *Z*_*α*_, and *Z*_*β*_ are reported in Table 4. As shown in Table 4, the variation of π* values between anisotropic and isotropic phases, called *Z*_π*_, exhibits its highest value for LC-III. This can be associated with the large Δ*ε* value as the main reason for the high dipolarity/polarizability ability, which remains when changing from anisotropic to isotropic state. On the other hand, LC-I shows a minimum π* value considering the sharp reduction of the dipolarity/polarizability property, whereas the phase transition occurred.

**Table 4:** Overall and typical matrix anisotropy polarity parameters obtained for the studied nematic LCs

Liquid crystal	*Z*_π*_	*Z*_*α*_	*Z*_*β*_	*Z*_o_	*Z* ^N^_o_
LC-I	0.01 ± 0.02	0.14 ± 0.02	0.01 ± 0.01	2.15 ± 0.02	0.06 ± 0.03
LC-II	0.05 ± 0.01	0.10 ± 0.02	0.02 ± 0.02	2.31 ± 0.01	0.07 ± 0.02
LC-III	0.08 ± 0.01	0.09 ± 0.01	0.04 ± 0.02	2.64 ± 0.02	0.08 ± 0.01

The obtained values of *Z*_o_ and *Z*^N^_o_ demonstrate their maximum and minimum values for LC-III and LC-I, respectively. These results can be conceived because of the strong dependence of the value of the overall matrix anisotropy on the mean dielectric constant in the LCs, which as a result increases the electrostatic interaction between the LC molecules. However, it is notable that in typical interactions, standing the functional groups in molecular structures of the LCs, the existing HBA and HBD capability, LC polarity, and structural steric effects could be affected by the matrix anisotropy effects.^21,22^ Nevertheless, values of *Z*_o_ for the studied LCs sorted as LC-III > LC-II > LC-I, according to their mean dielectric constant (*ε̄*) magnitudes. The same conclusion can be made considering the values of *Z*_o_ as strong dependence on *ε̄* in the comparison of LC-IV with MLC-2053 and MLC-2144 (ref. 43) by presenting the lower mean dielectric constant for LC-III (*ε̄* = 20.6) compared to *ε̄* = 23.7 and 22.1 for MLC-2053 and MLC-2144, respectively.^43^

To verify our statement regarding the importance of the mean dielectric constant magnitudes in the comparison of *Z*_o_ values instead of other physical factors, we compared the *Z*_o_ values of LC-I and ML-0643.^43^ As shown in Table 2, the Δ*ε* and Δ*n* values for LC-I were approximately similar to those of ML-0643 (Δ*ε* = 6.9 and Δ*n* = 0.103);^43^ on the other hand, the *ε̄* values for ML-0643 and LC-I are 5.6 and 6.46, respectively. Therefore, it is predictable that the *Z*_o_ value in ML-0643 shows a lower magnitude (2.11) than that of LC-I, and by observing their *Z*_o_ values our claim comes true.

### 3.5 Correlation of solvatochromic polarity parameters in the liquid crystals

There is no doubt there are many empirical solvent scales for the organic solvents, whereas the expansion of most solvent scales is limited by the inherent characteristics of the nominated reference process, such as chemical reactions between solute and solvent and solubility problems. Therefore, the determination of solvent parameters is excluded for important solvents. For this reason, the most comprehensive empirical solvent scales provided by the solvatochromic method can be considered one of the best and most reliable methods because of its easy measurement process. However, the solvent scales are widely used in the correlation analysis of solvent effects, including π*, *β*, and *α*, whereas the correlation analysis method is broadly measured for a large set of organic solvents.^14^ Accordingly, by defining the solvent polarity parameters, we were able to investigate the empirical solvent effects. In applying these parameters, it is implicitly presumed that the intermolecular interactions in the reference system used to develop a specific solvent scale are analogous to those in the system for the prediction of solvent effects. One of the most successful treatments was proposed by Kamlet–Taft^14,38^ for solvent effects by using a multiparameter equation called the linear solvation energy relationship. The multiparameter equation describes any solute property's variation with the solvent composition regarding a linear combination of the molecular parameters with π*, *β*, and *α*. To consider aspects of solvation, a multiparameter approach is used in the general form:^14,38^

9 *Y* = *Y*_0_ + *aα* + *bβ* + *s*π*

*Y* is the value of a solvent-dependent physicochemical property in a given solvent, and *Y*_0_ is a solute property in a given solvent or a hypothetical solvent where π* = *β* = *α* = 0. The coefficients of *a*, *b*, and *s* are considered solute-dependent but solvent-independent by the regression coefficients describing the sensitivity of property *Y* to the different solute/solvent interaction mechanisms.^14^ However, the use of single solvent parameters to predict solvent effects should be quite restricted. Indeed, using two parameters of the multiparameter approach serve as good approximations of solvent polarity for organic solvents.^14^ In particular, there are good linear correlations between empirical solvent polarity parameters was acceptable by proposing correlations between the *E*_T_(30) or *E*^N^_T_ parameters and the KAT parameters π* and *α* as determined by Marcus for isotropic solvents^14,54^ according to the following expressions:

10 *E*_T_(30) = 11.5π* + 15.2*α* + 31.2

11 *E*^N^_T_ = 0.36π* + 0.47*α* + 0.01

In spite of having a large set of these correlations for ordinary solvents, there is no evidence for the nematic LCs as anisotropic solvents. For this reason, this issue persuades us to make new correlations for *E*_T_(30) and *E*^N^_T_ parameters for the investigated LCs as specific media in the nematic state. We used the values of our previous results for the nematic LCs^21,22,43^ as well as from the present study: 15 LCs in total in the same experimental process. We obtained two excellent correlations of *E*_T_(30) and *E*^N^_T_ parameters, providing strong regressions and satisfactory correlations for the nematic LCs by:

12 *E*_T_(30) = (32.14 ± 0.70) + (13.42 ± 0.80)π* + (13.64 ± 0.90)*α*, standard error of the fitting = 0.38, *R*^2^ = 0.97

13 *E*^N^_T_ = (0.04 ± 0.02) + (0.42 ± 0.03)π* + (0.41 ± 0.02)*α*, standard error of the fitting = 0.01, *R*^2^ = 0.96

By observing these two correlation equations, no significant difference between π* and *α* parameters can be seen. Therefore, *E*_T_(30) and *E*^N^_T_ values are increased by the dipolarity/polarizability (π* values) and HBD (*α* values) abilities.

In the next step, we proposed special multiparameter correlations for providing a better quantitative description of the solvent effect by using the π*, *β*, and *α* parameters. However, various multiparameter correlations have been introduced for various solvent-dependent process polymers^34^ and solvents.^14^ By inspiration of these materials, we introduced a multiparameter correlation for the nematic LCs. By using the values of Table 5, satisfactory multiparameter correlations are obtained as:

14 *E*_T_(30) = (34.43 ± 1.30) + (17.83 ± 2.29)π* + (13.53 ± 0.89)*α* − (8.59 ± 1.50)*β*, standard error of the fitting = 0.37, *R*^2^ = 0.97

15 *E*^N^_T_ = (0.155 ± 0.07) + (0.64 ± 0.14)π* + (0.419 ± 0.03)*α* − (0.43 ± 0.2)*β*, standard error of the fitting = 0.01, *R*^2^ = 0.96

**Table 5:** The obtained solvatochromic parameters of nematic liquid crystals from the previous studies as well as present work for making the correlation between *E*_T_(30) and *E*^N^_T_ with the Kamlet–Abboud–Taft (KAT) polarity functions (*α*, *β*, and π*) at room temperature (25 °C)

Liquid crystal	π*	*α*	*β*	*E*_T_(30)	*E* ^N^_T_
1294-1b^22^	0.88	0.19	0.72	46.5	0.49
6CHBT^22^	0.82	0.08	0.65	44.7	0.44
MBBA^22^	0.80	0.08	0.68	44.3	0.42
7CP5BOC^22^	0.62	0.08	0.58	41.8	0.34
7CP7BOC^22^	0.61	0.07	0.60	41.5	0.33
5CB^21^	0.88	0.15	0.72	45.7	0.46
6CB^21^	0.87	0.14	0.72	45.4	0.45
E_7_ (ref. 21)	0.87	0.15	0.72	45.6	0.46
ML-0643 (ref. 43)	0.55	0.26	0.54	42.9	0.37
MLC-6292 (ref. 43)	0.56	0.33	0.55	43.66	0.40
MLC-2053 (ref. 43)	0.62	0.29	0.58	44.31	0.42
MLC-2144 (ref. 43)	0.81	0.35	0.68	48.2	0.54
LC-I	0.50	0.28	0.52	42.2	0.36
LC-II	0.46	0.49	0.49	43.01	0.45
LC-III	0.62	0.36	0.58	45.39	0.45

**Fig. 6:**
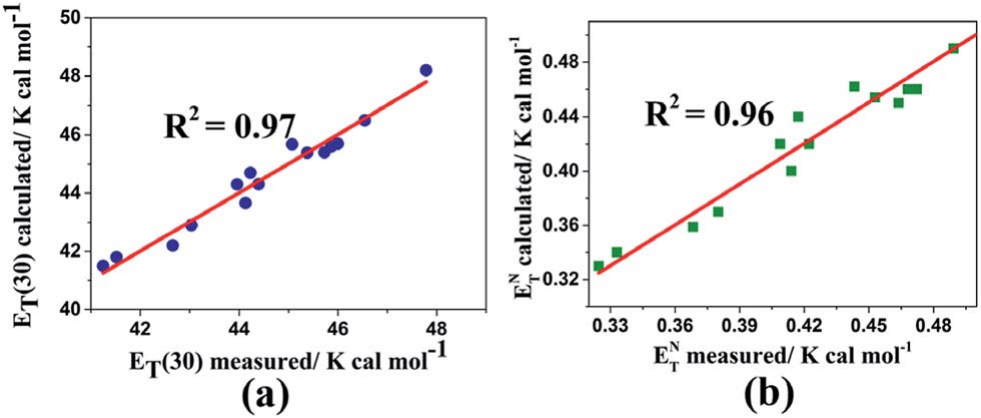
Calculated *versus* measured multiparameter correlations for (a) *E*_T_(30) and (b) *E*^N^_T_ using the solvatochromic polarity *α*, *β*, and π* parameters in the nematic liquid crystals using eqn (14) and (15), respectively.

Fig. 6 shows the measured and calculated of multiparameter correlations for *E*_T_(30) and *E*^N^_T_ values using the solvatochromic polarity *α*, *β*, and π* parameters. By comparing the *E*_T_(30) correlation between the two multiparameter equations (*i.e.*, (14) and (15)), it is recognizable that the *E*_T_(30) and *E*^N^_T_ values are increased by the dipolarity/polarizability (with large π* values) as the most significant and dominant factor. The negative sign of the *β* parameter based on the correlation equations can be expressed that the *E*_T_(30) and *E*^N^_T_ values decrease by HBA capacity. Because of the normalization of the π*, *β*, and *α* scales (from 0.0 to 1.0), the *a*/*s*, *b*/*s*, and *a*/*b* ratios are assumed to deliver quantitative measures of the relative contribution of the specified solvent parameters. Eqn (12) proposes that HBD capacities of LCs contribute a weaker effect on the *E*_T_(30) value than those of regular solvents because of the lower coefficient ratio *a*/*s* = 1.01 (derived from eqn (12)) than from using eqn (10) for solvents (*a*/*s* = 1.32).

However, these nematic LCs contribute equally to the correlations of both *E*_T_(30) and *E*^N^_T_. It is notable that because any correlation of solvent effects of a specific process with an SP parameter is a comparison with the effect of solvent on a reference process, we cannot expect these correlating parameters to be universally reliable for all types of solvent-sensitive processes. However, most of the existing empirical solvent scales exhibit good agreement with each other qualitatively and even quantitatively. Moreover, more nematic LC results must be determined and investigated to obtain an optimized relationship.

## 4 Conclusions

The solvent polarity parameters of unknown-molecular-structure LCs were determined by using spectroscopic measurements. We selected three low dielectric constants (Δ*ε*), with two of them having analogous and positive Δ*ε* values, in order to investigate the sensitivity of SP parameters, and one having a negative Δ*ε* value along with one high Δ*ε* to compare with the lower Δ*ε* values of LCs. The KAT polarity functions were determined at different temperatures and liquid crystal phases, isotropic and anisotropic.

The results of high *E*_T_(30) and *E*^N^_T_ values for these LCs, strong inter- and intra-molecular interactions in LC media, were confirmed in comparison with ordinary solvents. The large π* and *β* values in these LCs represent the effective polarity parameters as dipolarity/polarizability and basicity abilities, most likely because of the existing inherent dipolar properties of LCs and HBA groups in these LC compounds. Moreover, the determined π* and *β* values for the studied LCs showed higher magnitudes as compared with ordinary organic solvents at a similar dielectric constant.^14^ Thus, this result represents strong interactions between LC media and dipole solutes as well as HBD ability. By observing the temperature dependence of all SP parameters in the nematic and isotropic phases as well as the phase transition region, a slightly lower reducing slope in the nematic phase compared to the isotropic state can be perceived with increasing temperature. However, a sharp reduction from the nematic to the isotropic phase transition region was observed with increasing temperature. Therefore, reduction of the SP parameter values revealed a similar trend with other physical parameters with increasing temperature, in particular, in the nematic to isotropic phase transition area. Moreover, the SP functions provide quantitative values even in the isotropic phase, whereas the other macroscopic physical parameters vanish completely. Therefore, these parameters reveal their significance remarkably in this specific area and can even be employed in new applications such as optical and electro-optic switching, nonlinear optics and electro-optical Kerr effect in the isotropic phase of nematic LCs.

By considering this fact, the variations between SP parameters in the isotropic and anisotropic phases provide the structural balancing anisotropy feature for prediction of interactions and dipolarity/polarizability properties in the LC phase; overall and typical matrix anisotropy was determined for the investigated LCs by showing the LCs' polarity functions. The overall matrix anisotropy (*Z*_o_) values showed LC-III > LC-II > LC-I. The reason for this sequence is attributed to the sorting of the mean dielectric constant (*ε̄*) and dielectric anisotropy (Δ*ε*) values.

Finally, we introduced the linear solvation energy relationship for the empirical solvent scales (*i.e.*, *E*_T_(30) and *E*^N^_T_) with the KAT parameter sets for the nematic LC media for the first time. These relationships were well-established in both parameters (*i.e.*, using π* and *α*) and multiparameter (*i.e.*, using π*, *β*, and *α*) approaches. From the proposed correlation equations for the nematic LCs, it is understandable that for LCs as polar media, the effect of dipolarity/polarizability (π* value) is a more significant parameter than other KAT parameters on the *E*_T_(30) and *E*^N^_T_ values.

## Conflicts of interest

There are no conflicts to declare.

## Supplementary Material

## References

[cit1] Svedberg T. (1917). Colloid Polym. Sci..

[cit2] Haase W., Wedel H. (1977). Mol. Cryst. Liq. Cryst..

[cit3] Martire D. E., Blasco P. A., Carone P. F., Chow L. C., Vicini H. (1968). J. Phys. Chem..

[cit4] Emsley J. W. and Lindon J. C., NMR spectroscopy using liquid crystal solvents, Elsevier, 2013

[cit5] Lansac Y., Glaser M. A., Clark N. A., Lavrentovich O. D. (1999). Nature.

[cit6] Tilton M. , Liquid crystals: applications and uses, ed. B. Bahadur, World Scientific, Singapore, 1992

[cit7] Nakano T., Hirata H. (1982). Bull. Chem. Soc. Jpn..

[cit8] Ramamurthy V. (1986). Tetrahedron.

[cit9] Weiss R. G. (1988). Tetrahedron.

[cit10] Ramamurthy V. and Schanze K. S., Photochemistry of organic molecules in isotropic and anisotropic media, CRC Press; 2003

[cit11] Khoo I. C. , Liquid crystals: physical properties and nonlinear optical phenomena, John Wiley & Sons, New York, vol. 64, 2007

[cit12] Yang D. K. , Fundamentals of liquid crystal devices, John Wiley & Sons, New York, 2014

[cit13] Goodby J. , Gray G. W., Spiess H. W. and Vill V., Physical properties of liquid crystals, Wiley-VCH, Weinheim, Germany, 1999

[cit14] Reichardt C. and Thomas W., Solvents and solvent effects in organic chemistry, John Wiley & Sons, New York, 2011

[cit15] Jonquières A., Roizard D., Lochon P. (1994). J. Appl. Polym. Sci..

[cit16] Grunwald E., Winstein S. (1948). J. Am. Chem. Soc..

[cit17] Malavolta L., Oliveira E., Cilli E. M., Nakaie C. R. (2002). Tetrahedron.

[cit18] Reichardt C. (2008). Pure Appl. Chem..

[cit19] Bylińska I., Wierzbicka M., Czaplewski C., Wiczk W. (2014). RSC Adv..

[cit20] Kumari R., Varghese A., George L., Sudhakar Y. N. (2017). RSC Adv..

[cit21] Zakerhamidi M. S., Shahrabi S. (2013). Liq. Cryst..

[cit22] Zakerhamidi M. S., Shahrabi S. (2013). Liq. Cryst..

[cit23] Katritzky A. R., Fara D. C., Yang H., Tämm K., Tamm T., Karelson M. (2004). Chem. Rev..

[cit24] Reichardt C. (1979). Angew. Chem., Int. Ed..

[cit25] Fowler F. W., Katritzky A. R., Rutherford R. J. D. (1971). J. Chem. Soc. B.

[cit26] Dougherty R. C. (1975). Tetrahedron Lett..

[cit27] Hantzsch A. (1922). Ber. Dtsch. Chem. Ges..

[cit28] Reichardt C. (1994). Chem. Rev..

[cit29] Katritzky A. R., Fara D. C., Yang H., Tämm K., Tamm T., Karelson M. (2004). Chem. Rev..

[cit30] Brooker L. G. S., Keyes G. H., Heseltine D. W. (1951). J. Am. Chem. Soc..

[cit31] Kosower E. M. (1958). J. Am. Chem. Soc..

[cit32] Kosower E. M. , An Introduction to Physical Organic Chemistry, Wiley & Sons, New York, 1968

[cit33] Spange S., Keutel D., Simon F. (1992). J. Chim. Phys. Phys.-Chim. Biol..

[cit34] Spange S., Vilsmeier E., Fischer K., Reuter A., Prause S., Zimmermann Y., Schmidt C. (2000). Macromol. Rapid Commun..

[cit35] Buncel E., Rajagopal S. (1990). Acc. Chem. Res..

[cit36] Hirsch J. A. , Concepts in theoretical organic chemistry, Allyn and Bacon, Boston, MA, 1974

[cit37] Gutmann V. , The donor–acceptor approach to molecular interactions, Plenum Publ. Corp, New York, 1978

[cit38] Kamlet M. J., Abboud J. L., Taft R. (1977). J. Am. Chem. Soc..

[cit39] Taft R. W., Kamlet M. J. (1976). J. Am. Chem. Soc..

[cit40] Kamlet M. J., Taft R. (1976). J. Am. Chem. Soc..

[cit41] Sıdır Y. G., Sıdır İ. (2015). J. Mol. Liq..

[cit42] Ichikawa T., Fujimura K., Yoshio M., Kato T., Ohno H. (2013). Chem. Commun..

[cit43] Ranjkesh A., Hagh Parast M., Park J. S., Choi J. C., Zakerhamidi M. S., Kim H. R. (2017). Liq. Cryst..

[cit44] Wyatt V. T., Bush D., Lu J., Hallett J. P., Liotta C. L., Eckert C. A. (2005). J. Supercrit. Fluids.

[cit45] Nicolet P., Laurence C. (1986). J. Chem. Soc., Perkin Trans. 2.

[cit46] Kamlet M. J., Abboud J. L., Abraham M. H., Taft R. W. (1983). J. Org. Chem..

[cit47] Reichardt C. (2005). Green Chem..

[cit48] Lee J. M., Ruckes S., Prausnitz J. M. (2008). J. Phys. Chem. B.

[cit49] Gregory A. P., Clarke R. N. (2005). Meas. Sci. Technol..

[cit50] Maryott A. A. and Smith E. R., Table of dielectric constants of pure liquids, National Bureau of Standards, Gaithersburg MD, 1951

[cit51] Jessop P. G., Jessop D. A., Fu D., Phan L. (2012). Green Chem..

[cit52] Cerón-Carrasco J. P., Jacquemin D., Laurence C., Planchat A., Reichardt C., Sraïdi K. (2014). J. Phys. Org. Chem..

[cit53] Laurence C., Nicolet P., Dalati M. T., Abboud J. L., Notario R. (1994). J. Phys. Chem..

[cit54] Marcus Y. , The properties of solvents, John Wiley & Sons, Chichester, New York, 1998

